# Professor Doctor Marian Pardela In Memoriam Renowned Polish Surgeon and a Pioneer in The Field of Bariatric Surgery

**DOI:** 10.1007/s11695-021-05664-8

**Published:** 2021-08-31

**Authors:** W. Konrad Karcz, Barbara Osiadacz

**Affiliations:** 1 LMU Munich, Munich, Germany; 2 Poznań University of Life Sciences, Poznań, Poland


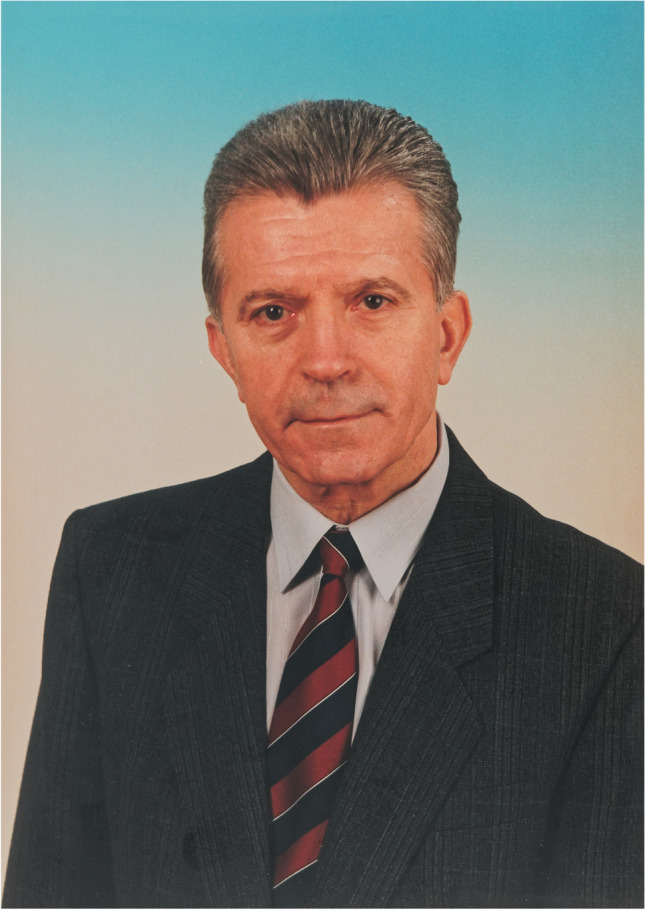
Professor Marian Pardela + , MD, died on January 31st, 2021, at the age of eighty-five. He was an outstanding surgeon and a renowned specialist in the gastrointestinal field, in particular, in liver, biliary tract, vascular, oncologic, and thyroid surgery. He was one of the pioneers of liver transplantation in Poland and devoted his professional life to the development of effective methods and tools, which made it possible to carry out innovative surgeries. Most of all, however, he was a precursor of surgical treatment of obesity in Poland and Europe and the founder of the school of surgical treatment of obesity in Poland, who dedicated his life to propagating the discipline as well as teaching it to others.

Marian Pardela was born on April 1st, 1935, in Skałat, Tarnopol province, Poland. During Second World War in 1940, his father was murdered in Ostashkov (Russia) that brought him with his mother and sister back to Upper Silesia, Poland, where, in 1951, he enrolled in Feldsher high school in Siemianowice. Three years later, he commenced his studies at the Medical Faculty of the Silesian Medical Academy in Zabrze (today: Medical University of Silesia). He graduated in 1961 and embarked on his clinical journey through several university hospitals in Zabrze, Katowice Ligota, and Bytom. Early on, he was mentored by outstanding surgeons and teachers: Prof. Józef Gasiński and Prof. Stanisław Szyszko (former students of Prof. Jan Glatzel, who continued the Vienna Surgical School of Prof. Johannes von Mikulicz-Radecki in Cracow and of course prolonged school of Prof. Ludwig Anton von Rydygier). Their mentorship helped shape and develop the ‘surgical character’ of Marian Pardela as he progressed along his professional path from assistant (1962) to professor (1992). In 1967, he received his doctoral degree based on his thesis entitled ‘The influence of mixed metal alloys on healing of bone tissue under experimental conditions’. He also gathered invaluable surgical experience working between 1972 and 1974 as the head of the surgical department of the Bombo Hospital in Tanga, Tanzania.

In 1972, he came across literature on surgical treatment of obesity for the first time. This encounter sparked his interest in the topic which, at the time, was an untrodden land in Poland. In 1976, he performed his first bariatric operation (JIB). He received habilitation in 1977 based on the thesis entitled ‘Effect of experimental intestinal bypass on functional and submicroscopic changes in the liver depending on the duration of observation’. He was one of the first people to note that intestinal exclusion by Payne and De Winde’s method could lead to severe damage of liver parenchyma and, based on this observation, he modified the technique (Pardela jejunoileostomy ‘45 × 15’ + VT) in 1985. In the same year, he became the head of the Department and Clinic of General and Vascular Surgery in Zabrze, which he would continue to lead until his retirement in 2005. Throughout that period, he successfully performed a broad spectrum of bariatric procedures: he was the first surgeon in Poland to introduce the VGB (in 1993), LAGB (1998), BPD, PBD-DS, BRYGBP (1999) and LRYGBP (2000). Thanks to his efforts, more than 1000 patients underwent bariatric surgery between 1977 and 2005.

Thanks to his initiative, the Outpatient Clinic for Surgical Treatment of Obesity in Zabrze was established in 1987 as the first institution of its kind in Poland. He cooperated with well-known bariatric surgeons, such as Prof. E. Mason, Prof. B. Husemann, Prof. N. Scopinaro, Prof. R. Weiner, Prof. M. Fried. As a result of his cooperation with Prof. Krystyna Żwirska-Korczala, in 2000, he founded the Association for the Prevention and Treatment of Obesity, Thyroid Diseases, and Associated Metabolic Disorders, which became a member of the IFSO in the consecutive year. In April 2002, under his direction, the first National Conference ‘Surgical treatment of obesity’ took place. In 2003, the Clinic was renamed to the Department of General and Bariatric Surgery in Zabrze — becoming the first obesity-dedicated center in Poland.

Marian Pardela retired in 2005, but did not resign from teaching and assisting his patients. Until 2019, he worked at the Oncologic and Reconstructive Surgery Clinic in the Maria Sklodowska-Curie Memorial Cancer Center and Institute of Oncology in Gliwice. Under his supervision, 28 students obtained their doctoral degrees, 19 fellows received specialization in surgery, four habilitated, and two were granted professorship. He published 262 scientific papers (of which 51 were dedicated to obesity) and 148 conference abstracts (72 on obesity). He presented 203 lectures at symposia and congresses and edited three monographs. An active innovator, he patented five devices of his own invention — two of which (Pardela retractor and Pardela stretcher) became widely used in the treatment of obese patients.

He received numerous awards and honors for his scientific and didactic achievements from the Polish Ministry of Health and Social Welfare, the Rector of the Silesian Medical Academy, and the Association of Polish Surgeons. He received many state distinctions, including the Officer's Cross of the Order of Polonia Restituta by the President of the Republic of Poland. Moreover, in recognition of his role as the President of the IFSO Member Society and his contribution toward the growth of the IFSO, he was granted the organization’s special award in 2009. Privately, he was passionate about sports: an excellent swimmer, a competitive tennis player, and a hobbyist sport shooter.

We will remember Professor Marian Pardela as a true inspiration for his students, a strong, unbreakable character capable of facing any adversity. He was a noble, extraordinarily modest, and sensitive person, truly committed to the Hippocratic Oath, and wholeheartedly dedicated to his patients. He was the leading authority in Poland in the field of surgical obesity treatment, who embodied the belief that, in his own words, ‘the bariatric treatment restores normality of life, and gives happiness again’.

He will be deeply missed

Prof. Dr. med. Dr. hc W. Konrad Karcz

Dr hab. biol. Barbara Osiadacz

